# Ebola virus disease outbreak; the role of field epidemiology training programme in the fight against the epidemic, Liberia, 2014

**DOI:** 10.11694/pamj.supp.2015.22.1.6053

**Published:** 2015-10-10

**Authors:** Mutaawe Lubogo, Bangure Donewell, Lucas Godbless, Sasita Shabani, Justin Maeda, Herilinda Temba, Theophil C Malibiche, Naod Berhanu

**Affiliations:** 1 Field Epidemiology & Laboratory Training Program, Uganda; 2 Field Epidemiology & Laboratory Training Program, Zimbabwe; 3 Field Epidemiology & Laboratory Training Program, Tanzania; 4 Field Epidemiology & Laboratory Training Program Ethiopia

**Keywords:** FETP, EVD, Ebola, Liberia

## Abstract

The African Field Epidemiology Network (AFENET) is a public health network established in 2005 as a non-profit networking alliance of Field Epidemiology and Laboratory Training Programs (FELTPs) and Field Epidemiology Training Programs (FETPs) in Africa. AFENET is dedicated to supporting Ministries of Health in Africa build strong, effective and sustainable programs and capacity to improve public health systems by partnering with global public health experts. The Network's goal is to strengthen field epidemiology and public health laboratory capacity to contribute effectively to addressing epidemics and other major public health problems in Africa. The goal for the establishment of FETP and FELTP was and still is to produce highly competent multi-disciplinary public health professionals who would assume influential posts in the public health structures and tackle emerging and re-emerging communicable and non-communicable diseases. AFENET currently networks 12 FELTPs and FETPs in sub-Saharan Africa with operations in 20 countries. During the Ebola Virus Disease (EVD) outbreak in West Africa, African Union Support for the Ebola Outbreak in West Africa (ASEOWA) supported FETP graduates from Uganda, Zimbabwe, Ethiopia and Tanzania for the investigation and control of the EVD outbreak in Liberia. The graduates were posted in different counties in Liberia where they lead teams of other experts conduct EVD outbreak investigations, Infection Control and Prevention trainings among health workers and communities, Strengthening integrated disease surveillance, developing Standard Operating Procedures for infection control and case notification in the Liberian setting as well as building capacity of local surveillance officers’ conduct outbreak investigation and contact tracing. The team was also responsible for EVD data management at the different Counties in Liberia. The FETP graduates have been instrumental in the earlier successes registered in various counties in Liberia in the control of the Ebola virus disease. Such efforts should be sustained by supporting local authorities develop strong health systems that are able to respond to epidemic of such magnitude in the near future.

## Introduction

Africa has been the epicenter of the most recent outbreaks of various diseases in the 21st century. One of such diseases has been the Ebola Virus Disease (EVD) that was officially notified by the World Health Organization (WHO) in Guinea in March 2014 [1]. The outbreak then spread to neighboring countries of Liberia, Sierra Leone, Mali and Nigeria in addition to North America and Europe. According to the Centre for Disease Control and Prevention (CDC) and the World Health Organization WHO Ebola Response plan, EVD has been responsible for 7,573 deaths out of the 12,386 confirmed cases in the three West Africa countries, Liberia being the most affected [2]. The rapid spread of Ebola among the three West African countries, namely Liberia, Sierra Leone and Guinea has been partly attributed to weak health systems, lack of skills and knowledge among health workers to manage the epidemic in addition to these countries having challenges with post conflict recovery [3]. In an effort to contain the frequently devastating epidemics in sub-Saharan Africa, the World Health Organization (WHO) Regional Office for Africa launched the Integrated Disease Surveillance and Response (IDSR) strategy in an effort to strengthen surveillance and response. The aim of such a strategy was to improve the surveillance of notifiable disease to provide information about disease outbreaks in various parts of the world for quick response. One of the key bottlenecks to this strategy was lack of human resources in many of the developing countries to support the roll out and the implementation of IDRS. African Field Epidemiology Network (AFENET) was established between 2005 and 2006 as a network of Field Epidemiology Training Programs (FETPs) and Field Epidemiology and Laboratory Training Programs (FELTPs) to support public health programs in different countries through disease surveillance and outbreak response in addition to surveillance systems strengthening in Africa [4]. This resulted from an expressed need to develop a network that would advocate for the unique needs of African FETPs and FELTPs, provide service to its membership, and through which programs could develop joint projects to address the public health needs of their countries [4–7].

**Figure 1:**
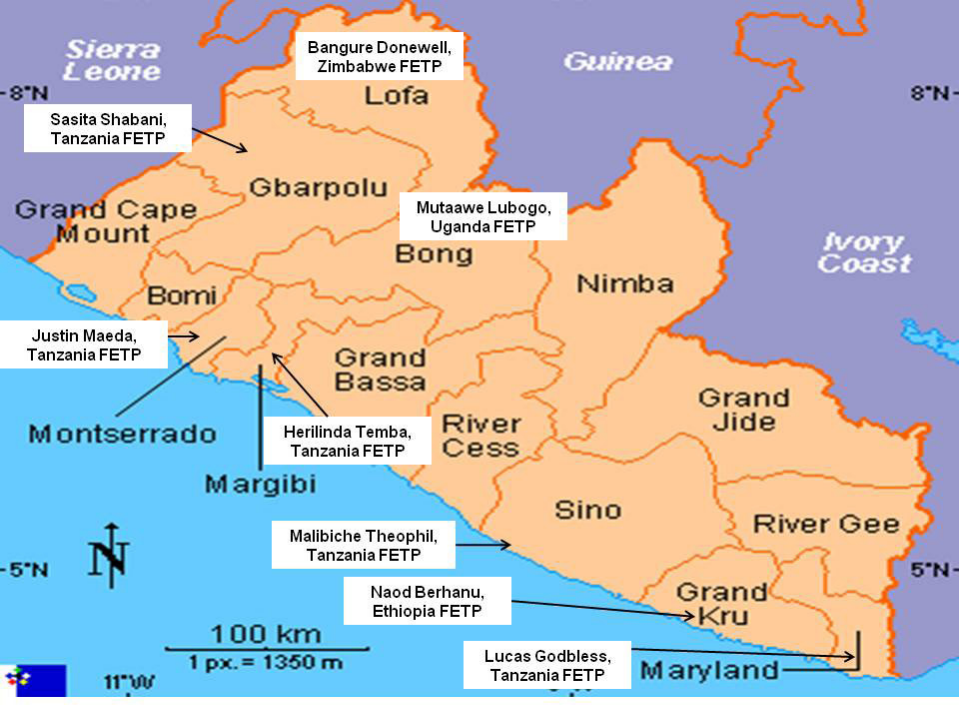
Deployment map for FETP Graduates for Ebola Viral Disease control in the counties, Liberia, 2014

AFENET currently networks 12 FELTPs and FETPs in sub-Saharan Africa with operations in 20 countries. AFENET has a unique tripartite working relationship with government technocrats from human health and animal sectors, academicians from partner universities, and development partners, presenting the Network with a distinct vantage point. Through the Network, African countries are making strides in strengthening their health systems through capacity building of public health professionals, resource mobilization and improving surveillance delivery through outbreak investigations and integrated disease surveillance[8]. Most of the FETP and FELTP graduates are highly competent multi-disciplinary professionals and have gone to assume leadership positions in both the public and private health sectors throughout Africa [9]. In the effort to control the spread of EVD in West Africa and the world, the African Union (AU) with support from CDC deployed 8 epidemiologists’ from Uganda, Zimbabwe, Ethiopia, and Tanzania to support Liberia in the prevention of further spread in the counties (Figure 1). This was because of the limited capacity of the West African nation to control and manage the epidemic without any external support [10].

## Roles of FETP graduates in the Liberia EVD response

### Capacity building of health workers in Ebola infection control and prevention (IPC)

One of biggest bottle necks in the control of the EVD is the lack of skills among health workers in the host country to manage Ebola outbreaks. This was evidenced when some of the health workers died in the line of their duty in Liberia, Sierra Leone and Guinea. This is partly due to the fact that this was the first time such an epidemic occurred in West Africa and lack of PPE to do barrier nursing by the health workers [11, 12]. The FETPs therefore embarked on training local health workers in IPC and use of Personal Protective Equipment (PPE). The trainings were conducted in Montserrado, Bong, Lofa, Margibi, Grand Kru and Sinoe with support form WHO and CDC. Training of health workers on Ebola infection control and prevention was not only important in averting possible further infections among health workers but also building local capacity manage any future outbreaks of EVD. These trainings were fielded based using practical guides and guidelines of EVD from WHO.

### Training of local communities in IPC and contact tracing

Studies have shown that EVD is spread from person to person when one comes in contact with body fluids of infected persons during caring for the sick, preparing dead bodies for burial and also through intimate relations [13, 14]. Most of the people in Africa usually offer treatment for their sick relatives and friends. Traditional rituals for the dead are also performed which could be potential source of infection. Such rituals include washing the bodies before burials and in some communities, this water is taken to be a sign of respect and avert curses form the dead [12]. Because of such practices that are vehicles for the rapid spread of EVD, it was important therefore to train different communities to stay safe in case one of their own contracted the virus. A number of communities were visited in the various Counties to do community sensitization and education about the spread of EVD and how to stay safe. In communities where general health community volunteers were identified, they were trained to notify the health facilities of any community death, suspected illnesses and also trained on training EVD contacts that had been identified by the case investigators. This was also important because most of the communities that were affected by EVD in rural Liberia were residing in very remote areas and were not accessible by road or Global System for Mobile Communications (GSMC) network. The community health volunteers would provide information by walking to the nearest health facility where the health workers would notify the County Health Teams for response. In some communities where GSMC existed, scratch cards were provide to General Community Health Volunteers (GCHVs) to report any of the suspected to the hot line at respective counties.

### Case investigations and rapid response

The graduates participated in the Investigations of EVD hot spots in Counties. The FETP graduate would lead a team of Epidemiologists, Psychosocial and social mobilization specialists investigate outbreaks in the remote villages of Bomo-Ta and Taylor-Ta. The rapid response team would conduct a meeting with the local communities to identify the suspects of any deaths recently in the community. The needs assessment would then be done ranging from clinical, social and psychosocial needs. The suspects would be evacuated to the nearest Community Care Centre and contacts will be listed in the due process. A community education session would be conducted bout Infection control and prevention. In any case the suspects were confirmed, such communities would be supported to be quarantined to prevent further spread. Most of these quarantines were community initiatives where no member of an affected community would be allowed to leave the town. In addition, visitors would not be allowed to visit the affected community. Community quarantines were always supervised by the local chiefs and the County Health Team and the local and international partners on EVD would support such communities with basic house hold items including food. The quarantine would be lifted after 21 days elapsed with no reported case form the data of the last case identified. In all the counties where the FETP graduates were deployed, they supported the integration of EVD control strategies in the Special Senatorial Electoral process that was held on the 20th of December 2014 in Liberia. The team ensured that all polling stations had hand washing facilities and thermoscan to screen for fever among the voters. However, no case was notified in all these counties on the polling day. In addition, after the team identified the gaps in the screening of travellers at check points, immigration centres and road blocks on all major high ways to counties were the FETP graduates had been posted, they also supported the screening of travellers for EVD. Reports had been received that cases and dead bodies were being transported form Montserrado one of the hardest hit counties to upcountry to seek treatment from traditional healers or get social support from their relatives. This was mostly implemented in Margibi and Bong counties.

### Development and dissemination standard operating procedures

During the month of October 2014, it was observed by the EVD Control Task Force in Montserrado County that there was a drastic drop in the number of cases reported to the local authorities. In addition, there had been rumors that some communities were concealing cases and conducting secret burials for the dead. This led to fears among the technical team of the potential surge in the number of EVD cases in Montserrado County which was one of the most affected counties in Liberia. The Epidemiologists from the African Union with support from the CDC therefore were tasked to establish the cause of the rapid decline in the number of cases that were notified during this period of time. The team therefore conducted a qualitative research in which community leaders and funeral directors were interviewed about the number of cases in their communities and the secrete burials that were alleged to have been conducted. The team found out that the decline in the number of EVD cases was real and this was attributed to behavior change among the communities due to intensified education and mobilization by the Liberia Ministry of Health and Social Welfare. In addition the leaders had observed improved response time by the burial team to pick up bodies form the communities. However, the communities expressed disappointment due of failure by the authorities to provide laboratory results on the cause of death of their relatives after testing for EVD. Based on this the epidemiologists developed Standard Operating procedures (SOPs) for death notifications and confirmation of death using the buccal swab technique by the burial teams and the funeral home directors. The SOPs that were developed by the epidemiologist were verified by the Liberian MoHSW and therefore distributed throughout the county.

### Integrated disease surveillance and response

One of the major components of the IDRS strategy was to use available resources do integrated disease surveillance so as to offer quick responses to all notifiable diseases. Liberia is one of the countries in West Africa that has had repeated outbreaks of vaccine preventable disease like polio and measles. In addition, Liberia is one of the countries were Lass fever is endemic has signs and symptoms similar to EVD. The FETP graduates therefore trained surveillance officers in Lofa and Bong counties conduct outbreak investigations for measles and Polio in 3 affected communities. Measles outbreak was detected in Voinjama District, Lofa County and an outbreak was declared by the County Health Team. As a result mass vaccination of those aged 6-59 months was conducted in Lofa County. In addition Lassa fever notification was intensified in all counties that are in the Lassa fever belt. According to the Bong county health team meeting, it was observed that four maternal mortalities had occurred between August and October 2014 partly because most of the attention and resources had been focused towards the control of EVD neglecting other health services. It was also observed that between August and October 2014, the number of Outpatient department (OPD) attendances in most of the health facilities in Bong had drastically dropped due to communities fearing contracting EVD form the health facilities. The FETP graduate in in Bong in collaboration with the Bong County Health Team, CDC and WHO instituted a surveillance for the notification of all pregnant women in remote communities at 32 weeks of Amenorrhea (WOA) and above for quick evacuation and care n health facilities till they delivered.

## Discussion

Since its inception, AFENET has responded to 133 suspected outbreaks ranging from environmental related outbreaks, vaccine preventable diseases, water and food borne, zoonoses, (including suspected virus hemorrhagic fevers) as well as neglected tropical diseases [5–7, 15, 16]. With its emphasis on one health approach of solving public health issues the program has trained physicians, veterinarians and laboratory scientists to work jointly on human, animal and environmental health issues. Residents have worked to identify risk factors of disease at the human animal interface for influenza, brucellosis, tick-borne relapsing fever, rabies, leptospirosis and zoonotic helminthic infections in Nigeria, Uganda, Ethiopia, Central Africa, Zimbabwe and Tanzania[4]. The skills and experiences acquired by these health professionals have been instrumental in the control of EVD in Liberia in addition to health systems strengthening through capacity building of the existing health workers and integrating surveillance of other diseases in the EVD surveillance activities [17, 18]. Graduates have worked in multidisciplinary teams with partners and host government to conduct EVD surveillance, outbreak investigations in Liberia [19]. Critical outcomes of these programs have therefore shown that development of public health leaders with core skills in integrated disease surveillance, outbreak investigation, vaccination campaigns, laboratory diagnostic testing, and epidemiological studies that address priority public health problems are key in addressing the emerging outbreaks in the 21st century [17, 20]. Because of the field based nature of the program, it was relatively easy for the graduates to acclimatize to the situation in West Africa partly because similar geographical and environmental factors that are determinants of EVD spread exist elsewhere in Africa where these graduates are working. In addition all of the graduates were familiar with the African social cultural set up hence it was easy to build confidence with the local leadership and communities so as to establish networks for case notification, contact tracing and quarantining affected communities.

The deployment of FETP graduates in the EVD outbreak in West Africa was the first of its kind assignment to be undertaken by the African Union. AU has a long history of offering military assistance to countries like Somalia and Sudan. The expansion of the AU mandate to support outbreaks and emergence response elsewhere on the continent is a positive step forward in preparing the continent for any future outbreaks. Despite these achievements a number of challenges were encountered by the FETP graduates in line of their duties. This being the first of deployment by the AU, a lot of logistics challenges could not be avoided partly because AU did not have prior experience in emergency response. Furthermore, because of the many needs encountered in the rural communities ranging from lack of skilled local health worker force and a relatively weak health system, it became difficult to define the scope of operation [21, 22]. In Counties like Sinoa and Lofa the FETP graduates had to take on a number of other roles not related to EVD, hence reducing their effectiveness as far as disease surveillance is concerned. The FETPS also faced a lot of hardship to conduct investigations in remote communities especially in Bong and Lofa. This was due to the fact that most of the affected communities in these counties were in remote areas that were not accessible by road and GSM network. The FETPS however endured the hardship of walking for long hours in search of cases and once there, community linkages were established to offer any information in time for any suspected cases. In an effort to strengthen the surveillance and outbreak investigations and response, countries that have been worst hit by EVD should be considered for FETP program set up to train local health workers in surveillance systems and also provide them with skills for future response. This could be done through onsite mentorship of health workers by the existing epidemiologists. The group also recommends implementation of studies aimed to understanding the dynamics of this EVD outbreak in West Africa in addition to studying the potential host of the EVD virus to offer health professionals with relevant knowledge of how to tackle the epidemic. In addition studies of possible vaccine trials for the virus hemorrhagic fevers should be expedited given the case fatalities associated with the EVD.

## Conclusion

FETP provides graduates with skills to respond to disease outbreak investigation and surveillance in the African context. The skills acquired by graduates have been used in the control of EVD in West Africa to avert further spread among the general population and health workers. However, there is need for continued support from both development partners and African governments to uphold the highlighted successes and to overcome looming challenges to advancement of field epidemiology implementation in sub-Saharan Africa.
